# Effectiveness of a multicomponent intervention to face the COVID-19 pandemic in Rio de Janeiro’s favelas: difference-in-differences analysis

**DOI:** 10.1136/bmjgh-2022-009997

**Published:** 2023-05-30

**Authors:** Amanda de Araujo Batista-da-Silva, Camila Barros Moraes, Helena Rodrigues Bozza, Leonardo dos Santos Lourenço Bastos, Otavio T Ranzani, Silvio Hamacher, Fernando Augusto Bozza

**Affiliations:** 1 Department of Industrial Engineering, Pontifical Catholic University of Rio de Janeiro, Rio de Janeiro, Brazil; 2 Tecgraf Institute, Pontifical Catholic University of Rio de Janeiro, Rio de Janeiro, Brazil; 3 Associação Redes de Desenvolvimento da Maré, Rio de Janeiro, Brazil; 4 Federal University of Rio de Janeiro, Rio de Janeiro, RJ, Brazil; 5 Barcelona Institute for Global Health, Barcelona, Spain; 6 University of Sao Paulo Hospital of Clinics, Sao Paulo, Brazil; 7 D'Or Institute of Research and Education, Rio de Janeiro, RJ, Brazil; 8 National Institute of Infectious Diseases Evandro Chagas, Oswaldo Cruz Foundation (FIOCRUZ), Ministry of Health, Rio de Janeiro, RJ, Brazil

**Keywords:** COVID-19, public health, control strategies, infections, diseases, disorders, injuries, intervention study

## Abstract

**Introduction:**

Few community-based interventions addressing the transmission control and clinical management of COVID-19 cases have been reported, especially in poor urban communities from low-income and middle-income countries. Here, we analyse the impact of a multicomponent intervention that combines community engagement, mobile surveillance, massive testing and telehealth on COVID-19 cases detection and mortality rates in a large vulnerable community (*Complexo da Maré*) in Rio de Janeiro, Brazil.

**Methods:**

We performed a difference-in-differences (DID) analysis to estimate the impact of the multicomponent intervention in *Maré,* before (March–August 2020) and after the intervention (September 2020 to April 2021), compared with equivalent local vulnerable communities. We applied a negative binomial regression model to estimate the intervention effect in weekly cases and mortality rates in *Maré*.

**Results:**

Before the intervention, *Maré* presented lower rates of reported COVID-19 cases compared with the control group (1373 vs 1579 cases/100 000 population), comparable mortality rates (309 vs 287 deaths/100 000 population) and higher case fatality rates (13.7% vs 12.2%). After the intervention, *Maré* displayed a 154% (95% CI 138.6% to 170.4%) relative increase in reported case rates. Relative changes in reported death rates were −60% (95% CI −69.0% to −47.9%) in Maré and −28% (95% CI −42.0% to −9.8%) in the control group. The case fatality rate was reduced by 77% (95% CI −93.1% to −21.1%) in *Maré* and 52% (95% CI −81.8% to −29.4%) in the control group. The DID showed a reduction of 46% (95% CI 17% to 65%) of weekly reported deaths and an increased 23% (95% CI 5% to 44%) of reported cases in *Maré* after intervention onset.

**Conclusion:**

An integrated intervention combining communication, surveillance and telehealth, with a strong community engagement component, could reduce COVID-19 mortality and increase case detection in a large vulnerable community in Rio de Janeiro. These findings show that investment in community-based interventions may reduce mortality and improve pandemic control in poor communities from low-income and middle-income countries.

**WHAT IS ALREADY KNOWN ON THIS TOPIC**

- During the COVID-19 pandemic, inequalities in access to healthcare resources have contributed to the higher burden of cases and death in socially vulnerable communities globally.
- Few interventions at the community level addressing the control of transmission and better COVID-19 case management have been implemented and evaluated, especially in poor, high-dense urban territories from low-income and middle-income countries.

**WHAT THIS STUDY ADDS**

- A multicomponent intervention combining community engagement strategies, mobile surveillance, massive testing and telehealth was implemented in a large vulnerable community composed of 16 favelas with 140 000 inhabitants in Rio de Janeiro, Brazil, to face the COVID-19 pandemic.
- The intervention reduced 46% (95% CI 17% to 65%) of weekly reported deaths and increased 23% (95% CI 5% to 44%) of reported COVID-19 cases.

**HOW THIS STUDY MIGHT AFFECT RESEARCH, PRACTICE OR POLICY**

- A complex intervention integrating new health surveillance and care models to tackle epidemics in poor urban communities could increase case detection and reduce mortality.
- These results might support future actions to establish more equitable epidemic responses, especially regarding access to the health system and care for socially vulnerable populations.

## Background

The COVID-19 pandemic has increased and exacerbated inequalities in almost every aspect of human life: access to healthcare and education, jobs and incomes, and technologies such as vaccines or digital resources. Socially vulnerable populations were significantly affected by the pandemic, reflected in higher rates of severe COVID-19 cases and deaths,^1 2^ and increased poverty and food insecurity.^3 4^ The World Bank estimated that the pandemic resulted in 97 million more people in poverty in 2020.^5^ Poverty has increased across all regions, particularly sub-Saharan Africa and Latin America.

Latin America has been an unequal region in the world (measured by the Gini Index), even before the onset of the COVID-19 pandemic. The pandemic profoundly affected Brazil, with around 30 million reported cases and more than 650 000 deaths at the beginning of 2022, resulting in political, social, economic and sanitary crises. The country has been vulnerable in several dimensions since its socioeconomic situation worsened in 2015, interrupting a trend of reduction in income inequality from the early 2000s.^6^ In December 2020, 55% of the Brazilian population was in a situation of food insecurity (116.8 million), and 19% were hungry (40.3 million),^7^ many of them living in poor urban communities (*favelas*) or peripheries of large metropoles.

*Favelas*, usually translated as slums or informal settlements, emerged in the mid-twentieth century during the country’s rapid urbanisation. Several times, *favelas* are identified with poor quality housing, a degraded environment and limited access to public health, education or sanitation services. Additionally, these territories have been associated with the presence of armed groups (gangs or militias) and the perception of insecurity.^8 9^ However, unlike the usual perspective, *favelas* are vibrant places with a robust civil society, and many have strong traditions of activism and self-organisation. Their residents have fought for better social conditions with influential and successful community-based social organisations, while there is the inaction of governments. During the pandemic, these regions were particularly affected.^3 10–12^ Public health discussions on whether the local population would be able to adhere to social distancing and isolation, as recommended by the authorities, have been present since the beginning of the pandemic.^13^

Although much has been said about inequity in access to health resources during the pandemic, few interventions to control transmission, testing and clinical treatment of COVID-19 cases have been evaluated at the community level,^14–16^ especially in poor, high-density urban communities from low-income and middle-income countries.^17 18^

The ‘Complexo da Maré’ is one of the largest favela complexes in Rio de Janeiro, Brazil, and was dramatically affected by the pandemic at the beginning, with mortality rates due to COVID-19 worse than the city’s average. Although neglected by the government, Maré also has a long history of mobilisation for civil rights and strong civil society organisations.^19^

Here, we aimed to estimate the impact of a multicomponent intervention in Maré combining community engagement strategies, mobile surveillance, testing, and telehealth on COVID-19-reported cases and deaths.

## Methods

### Setting

*‘Complexo da Maré*’ is the ninth most populated district and the largest vulnerable community in Rio de Janeiro. Maré has approximately 140 000 residents^20^ distributed in 16 favelas and occupies an area of 5 km².^21^ Maré has a low Human Development Index (HDI), ranked 123rd out of Rio’s 126 neighbourhoods, with an average HDI of 0.686.^21^

### The multicomponent intervention

The multicomponent and complex intervention is composed of four pillars: (A) Communication and community engagement, (B) Surveillance, (C) Healthcare, and (D) Management. This programme was operationalised by several players, as described below.

The initiative *‘Conexão Saúde: de olho na Covid’* was developed by a coordinated action of six institutions/initiatives (*Fundação Oswaldo Cruz, Redes da Maré, Dados do Bem (DdB), SAS Brasil, União Rio, and Conselho Comunitário de Manguinhos*, description in the online supplemental material) whose organisational arrangement included public and private sectors, academia, and non-governmental organisations. *Redes da Maré* is a civil society local organisation that participated in all states of this intervention, including its design, reporting and dissemination. The objective was to provide information, surveillance and health services to reduce the impact of the COVID-19 pandemic.

10.1136/bmjgh-2022-009997.supp1 Supplementary data

**Figure 1:**
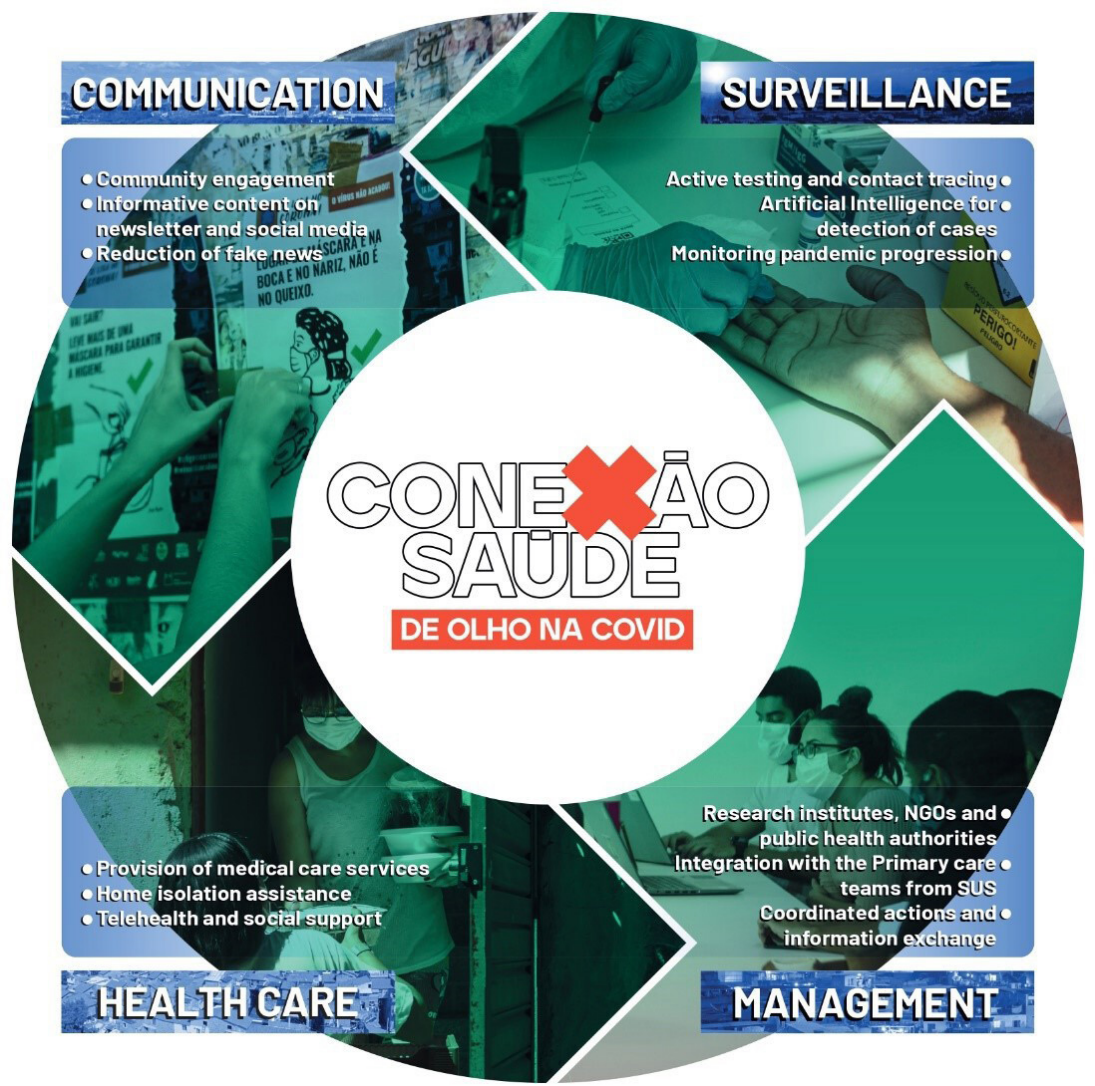
Overview of the integrated model for healthcare and surveillance in *favelas.* NGO, Non-Governanmental Organisation; SUS, Brazil's Unified Health System (*Sistema Único de Saúde)*.

*‘Conexão Saúde’* was designed as a multicomponent intervention. It proposes an integrative and participatory model of health surveillance and care by employing new technologies to expand access to healthcare and enable fast and effective responses to the population’s demands. Since September 2020, the multicomponent intervention has combined three main healthcare activities: (1) Massive testing promoted and managed by mobile technology, (2) Telehealth and (3) Home isolation with social support (figure 1).

#### Communication and community engagement

The communication strategy aimed to provide information about progression of the COVID-19 pandemic, especially the cases and deaths in the community, and protection strategies to orientate the residents. A team of local mobilisers was established to advise and engage residents in the services offered by the intervention. The team was trained to inform using adequate language to answer questions for a better understanding, thus reducing the diffusion of myths and fake news. Several community engagement approaches were implemented: visits to houses, work with primary healthcare units (*Unidade Básica de Saúde*) and residents' associations, distribution of hand sanitisers, masks and flyers, as well as placing banners and posters on the main streets of the *favelas* in *‘Complexo da Maré’ (Maré*). Overall, 72 000 informative folders about the project’s actions, 20 000 pamphlets about mobile testing, 1000 posters, 1700 copies of the Safe Home Isolation Guide, and 28 street banners were produced and distributed, including the 21 totems of hand sanitiser placed in the community. Furthermore, information about the pandemic was published weekly in newsletters and social media posts.^22^

#### Surveillance

The surveillance approach sought to control the community SARS-CoV-2 transmission and mitigate risks and health effects caused by COVID-19 mainly via testing activity. After July 2020, an independent and broad testing strategy was started in the community, including a testing centre and three mobile sites in different locations in the territory. In order to support the surveillance process, the intervention provided molecular and serological tests, free of charge, associated with a mobile app system, DdB.^23^

The testing enabled the identification of symptomatic cases and contacts through a mobile application. To become eligible for a test, the user registers in the app, through their phone or one available in the testing tents, and answers a self-assessment questionnaire with information about previous health conditions and symptoms associated with COVID-19. Using these data, an artificial intelligence (AI) algorithm calculated the probability of being infected with SARS-Cov-2.^23^ Users with a high infection risk were scheduled for testing in one of the sites, and the result became available in the app within the next 48 hours. In case of a positive outcome, the app requested the user to inform five people they had been in contact with within the previous days who might also be infected (contact tracing). Then, these contacts are invited to test. The positive results were notified to the Epidemiological Surveillance Information Systems (SIVEP-Gripe/e-SUS *Vigilância Epidemiológica (*VE)),^24 25^ Brazil’s nationwide surveillance database for COVID-19. DdB also has a back-office interface with reports, maps and administrator activities. The screening through the AI algorithm provided insightful information to understand the virus propagation rate and disease clusters, reducing the under-reporting of cases in *Maré*. Data from the app or the AI algorithm was anonymised, following guidelines for data management from the Brazilian General Data Protection Regulation (*Lei Geral de Proteção de Dados*).

#### Healthcare

The intervention intended to increase access to clinical monitoring and remote healthcare for suspected and confirmed COVID-19 cases in *Maré*. Telehealth was conducted by the *‘SAS Brasil*’ telemedicine programme,^26^ a non-profit organisation aiming to provide medical care through teleconsultations to low-income populations. Since two telemedicine booths were installed locally, residents without a smartphone could also receive assistance.

The home isolation programme assisted residents who have been exposed to or infected by COVID-19 (positive test). Mobilisers identified these residents and invited them to join the programme. Initially, the social team applied a questionnaire to understand the families’ demands and difficulties in carrying out adequate home isolation. Cleaning, meal and protection kits were delivered according to each family’s necessity. Oximeters were also provided to monitor the cases. The isolation programme, combined with telehealth, offered medical and psychological support during the isolation period. In addition, each participant received a guide produced by experts with instructions on avoiding household infection.

The initiative aided the follow-up of home-isolated patients with COVID-19 by monitoring the progression of the disease and referring the patient to high-complexity attendance when necessary, associated with social support, a critical factor for adhesion to the programme. Besides medical care directed to patients with COVID-19, the population had access to 22 medical specialties and a multidisciplinary team of nurses, psychologists and physiotherapists. This strategy contributed to supplying repressed healthcare demands for other diagnostics also.

#### Management

The institutions involved in the conduction of the intervention constituted a steering committee. They conducted weekly meetings in which representatives quickly made decisions and adjustments in the planning according to the pandemic’s dynamic.

Based on an integrated model of action and exchange of information, the multicomponent intervention conducted a progressive integration with the local primary care facilities. The initiative supported the healthcare centres overwhelmed during the pandemic through testing and telehealth activities.

### Study design

**Figure 2:**
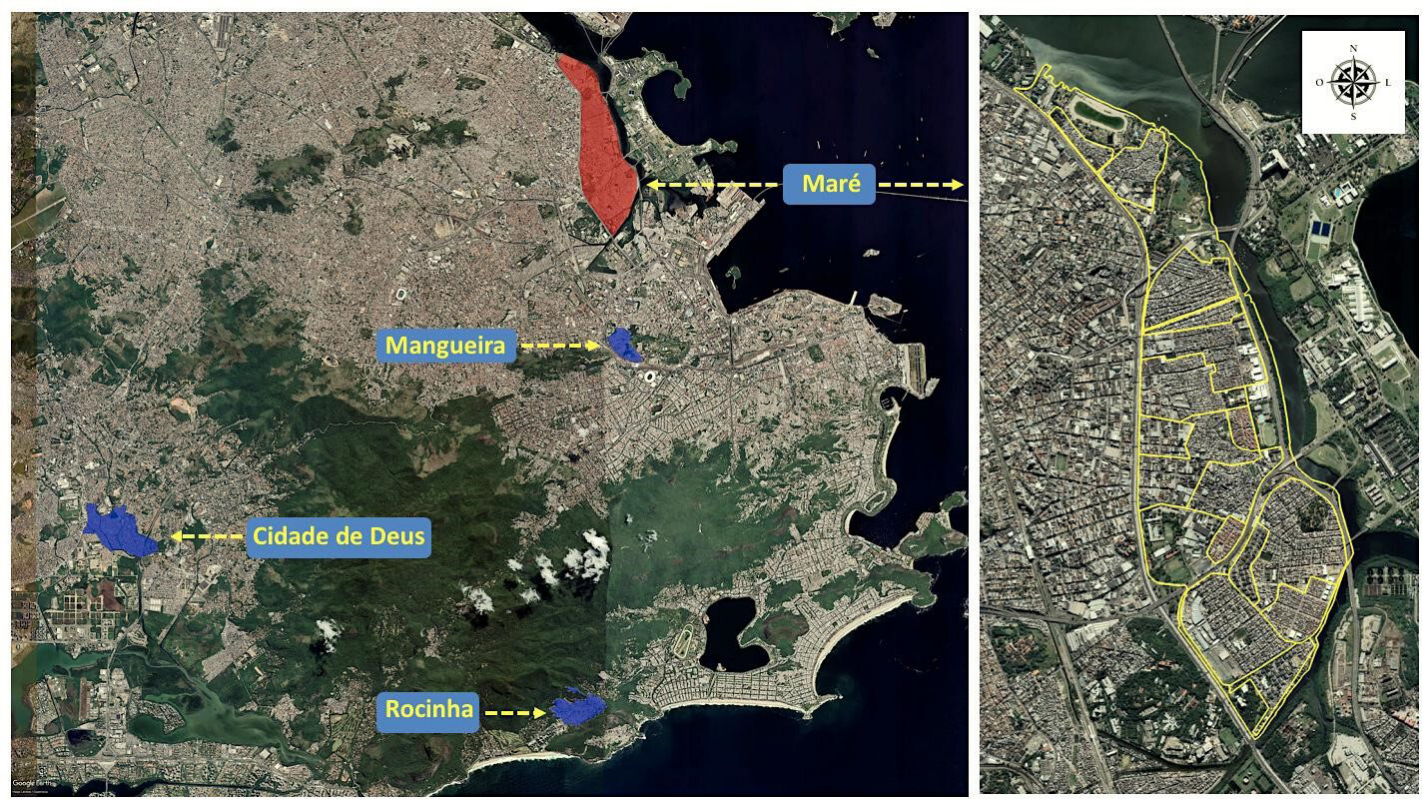
Map of Rio de Janeiro municipality. The red region delimits the *‘Complexo da Maré*‘—intervention group—and the blue regions delimit Rocinha, Cidade de Deus and Mangueira *favelas*—control group. The zoomed-in scale on the right shows the division of 16 favelas in the Maré region.

We performed a comparative before-and-after evaluation design using difference-in-differences (DID) analysis to estimate the impact of the multicomponent intervention in *‘Complexo da Maré*’ from March 2020 to April 2021. We considered *‘Maré’* as the intervention group in which the multicomponent intervention was applied. The ‘control’ was composed of the combination of the other three largest vulnerable communities in Rio de Janeiro*—Rocinha*, *Cidade de Deus* and *Mangueira*. We chose these areas that combined would be comparable to population size, density, socioeconomic indicators and overall features from Maré. Additionally, they are spatially non-contiguous, which decreases the potential contamination of the intervention across favelas. Their added population and socioeconomic indicators (Social Progress Index, HDI and income per person) are comparable to *Maré* (figure 2, online supplemental table A).

Preparations for the multicomponent intervention began in July. In September 2020, the integrated model for healthcare and surveillance was implemented, with all the strategies being executed simultaneously. We evaluated the effect of the three strategies combined into a single multicomponent intervention. Hence, our study considered the ‘before intervention’ period from March 2020 to August 2020 and the ‘after intervention’ from September 2020 to April 2021, previous to the vaccination mass campaign in *Maré*.^27^

### Data source and study population

We extracted the notification of COVID-19-reported cases and deaths from an open access database provided by the Rio de Janeiro municipality.^28^ The data are comprised of confirmed SARS-CoV-2 cases registered at the individual level since January 2020 in the Influenza Epidemiological Surveillance Information Systems: SIVEP-Gripe (*Sistema de Informação de Vigilância Epidemiológica da Gripe*)^24^ and e-SUS VE.^25^ We selected cases and deaths by place of residence from each favela analysed in the study*—Maré, Rocinha, Cidade de Deus* and *Mangueira*.

Regarding the data obtained after the intervention, we collected information on positive and negative tests taken by *Maré* residents from the DdB app from July 2020 to April 2021.^23^ All users provided informed consent for use of de-identified data for non-commercial research upon registration in the app. All answers were optional. The home isolation programme data were collected using structured questionnaires at two different time points: upon enrollment in the program and completion of the quarantine period. This data collection took place from September 2020 to April 2021. In addition, registers of appointments performed from July 2020 to April 2021 in the territory were made available by the *‘SAS Brasil’* organisation.

### Patient and public involvement

Community members were involved in the research’s design, conduct and dissemination plans of the project. For the intervention analysis, de-identified, aggregate data were used. In addition, the study also used a secondary data set that is publicly available. The research findings have been shared at stakeholder meetings, seminars, social media platforms and published reports.

### Measurements and outcomes

The primary outcome was age-sex standardised rates of cases and deaths per 100 000 population. Secondary outcomes included the age-sex standardised case fatality rate and positivity rates. Standardised rates were adjusted by age and sex using the Rio de Janeiro municipality population as the reference. Other variables enabling measuring the intervention’s impact were analysed, such as the number of tests performed, the number of appointments and the individuals included in the isolation programme. Outcomes were assessed before and after the onset of the multicomponent intervention for the intervention and control groups.

### Data analysis

We evaluated the progression of the COVID-19 pandemic in *Maré* during the study period. We described data using means and SD or median and IQRs for continuous variables, and frequencies and proportions for categorical variables. We analysed complete case data, and no missing value imputation was made.

We performed a twofold analysis to estimate the effect of the multicomponent intervention (all strategies combined) on the progression of cases and deaths per 100 000 population in *‘Complexo da Maré’*. First, we compared the relative changes in reported COVID-19 cases and deaths per 100 000 population and the case fatality rates between *Maré* and the control group before and after the onset of the multicomponent intervention.

Second, we conducted a DID analysis to evaluate the impact of the multicomponent intervention on the weekly progression of cases and mortality rates per age and biological sex. DID is a causal modelling approach used for impact evaluation by comparing treatment and control groups, before and after an intervention, under non-experimental settings (eg, non-randomised data).^29 30^ We obtained weekly reported cases and death rates in the intervention group (*Maré*) and the control group. The temporal indicator of the intervention period was the epidemiological week 36/2020 (30 August to 5 September), which represents the onset of the multicomponent intervention when the three components were simultaneously in place. To estimate the intervention effect size, we modelled data using a negative binomial regression model. The response variable was the outcome rate, and the covariates were the intervention/control group, the before-and-after intervention indicator, their interaction, the age group and the sex (more details are in online supplemental material sMethods). The intervention effect was calculated as the exponentiated coefficient of the interaction term, defined as the ratio of rate ratios (RRR).^31^

Finally, we evaluated the outputs of each component as a report of the joint actions from the intervention. We calculated the total number of test results, the characterisation of isolation programme participants and the number of appointments performed by telemedicine. We considered the significance level of 0.05 for statistical tests. All analyses were done in R V.4.0.2.

## Results

**Figure 3:**
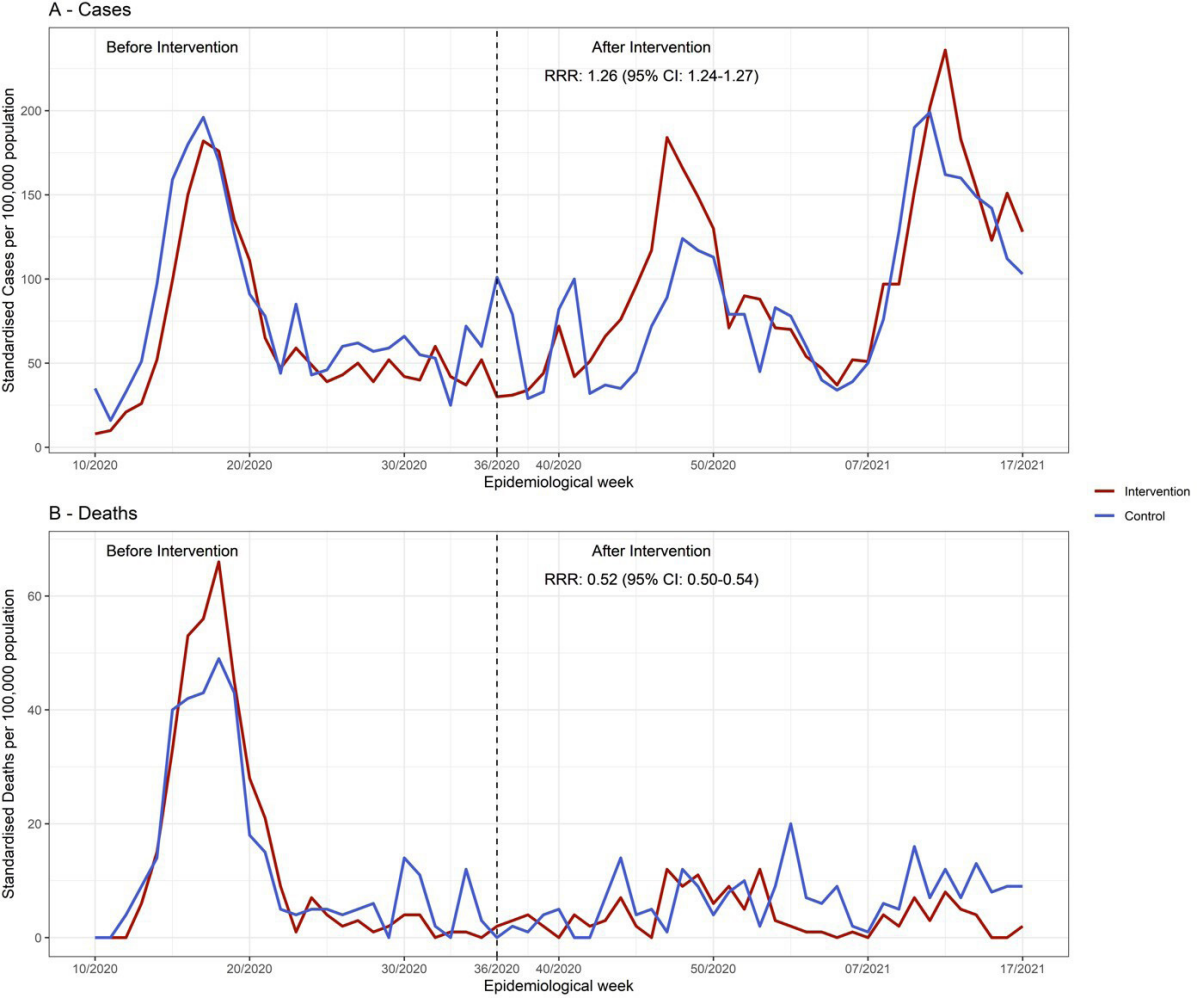
Estimated intervention effect in the progression of COVID-19 standardised (A) cases and (B) deaths per 100 000 population. Estimates of the ratio of rate ratios (RRR) were obtained from the Poisson regression model using difference-in-differences analysis. The intervention group was Maré, and the control group was jointly the favelas Rocinha, Cidade de Deus and Mangueira. The x-axis denotes the epidemiological week of (A) symptoms onset and (B) outcome. The dashed line represents the intervention onset. Data were obtained from the Surveillance Information Systems (SIVEP-Gripe/e-SUS VE). Rates were adjusted by age and sex using the Rio de Janeiro municipality population as a reference.

Between March 2020 and April 2021, 313 474 confirmed cases of COVID-19 and 26 613 deaths were notified in Rio de Janeiro municipality, with 4967 cases and 279 deaths reported from *‘Complexo da Maré’*. We analysed the progression of the pandemic and outcomes before and after the onset of the multicomponent intervention (table 1, figure 3). Before the intervention (March 2020 to August 2020), *Maré* presented lower rates of reported COVID-19 cases compared with the other *favelas* combined (control group) (1373 vs 1579 standardised cases per 100 000 population). The number of reported deaths was comparable between *Maré* and the control group (309 vs 287 standardised deaths per 100 000 population). However, *Maré* displayed a higher case fatality rate in this period (13.7% vs 12.2%). The performance of each favela that composes the control group is indicated in online supplemental table B.

**Table 1:** Relative change of pandemic indicators before (March 2020 to August 2020) and after (September 2020 to April 2021) the intervention period comparing the intervention (Maré) and control (Rocinha, Cidade de Deus, Mangueira) groups

	Intervention group (Maré)			Control group (Rocinha, Cidade de Deus, Mangueira)		
	Before intervention	After intervention	Relative change	Before intervention	After intervention	Relative change
Cases	1366	3470	154% (138.6% to 170.4%)	1528	2938	92% (80.8% to 104.5%)
Standardised * cases per 100 000	1373	3076	124% (110.2% to138.8%)	1579	2804	78% (67.0% to 88.9%)
Average standardised cases/100 000 per week †	53 (17.5)	88 (17.7)	66% (18.1% to133.5%)	61 (17.6)	80 (16.4)	31% (-6.0% to 83.0%)
Deaths	199	80	−60% (−69.0% to −47.9%)	188	136	−28% (−42.0% to −9.8%)
Standardised deaths per 100 000	309	119	−62% (−68.8% to −52.4%)	287	211	−27% (−38.5% to −12.2%)
Average standardised deaths/100 000 per week †	12 (7)	3 (1)	−75% (−92.9% to −11.4%)	11 (5.2)	6 (1.5)	−46% (−79.8% to 47.5%)
Standardised case fatality ratio	13.7	3.2	−77% (−93.1% to −21.1%)	12.2	5.9	−52% (−81.8% to 29.4%)

*Standardised rates were adjusted by age and sex.

†Mean (SD).

When we analysed the demographic characteristics of deaths reported before and after intervention comparing Maré to the control group (online supplemental table C), we observed a higher relative decrease in reported deaths in Maré in all groups independently of age or sex (online supplemental table D).

Comparing before and after the intervention, *Maré* displayed a 124% ((95% CI 110.2% to 138.8%), 1373 vs 3076 standardised cases per 100 000 population) increase in rates of reported standardised cases, and the control group showed a 78% increase ((95% CI 67.0% to 88.9%), 1579 vs 2804 per 100 000 population). The relative change in reported death rates was −62% ((95% CI −68.8% to −52.4%), 309 vs 119 standardised deaths per 100 000 population) in *Maré*, whereas within the control group, the change was −27% ((95% CI −38.5% to −12.2%), 287 vs 211 standardised deaths per 100 000 population). In addition, the case fatality rate reduced by 77% (95% CI −93.1% to −21.1%, 13.7% vs 3.2%) in *Maré* and 52% (95% CI −81.8% to −29.4%, 12.2% vs 5.9%) in the control group. As a result, the multicomponent intervention reduced 46% (RRR 0.54; 95% CI 0.35 to 0.83) the reported mortality rates per week in *Maré* compared with the control group. Furthermore, the number of reported cases per week increased by 23% (RRR 1.23; 95% CI 1.05 to 1.44) in *Maré* after the intervention onset.

**Figure 4:**
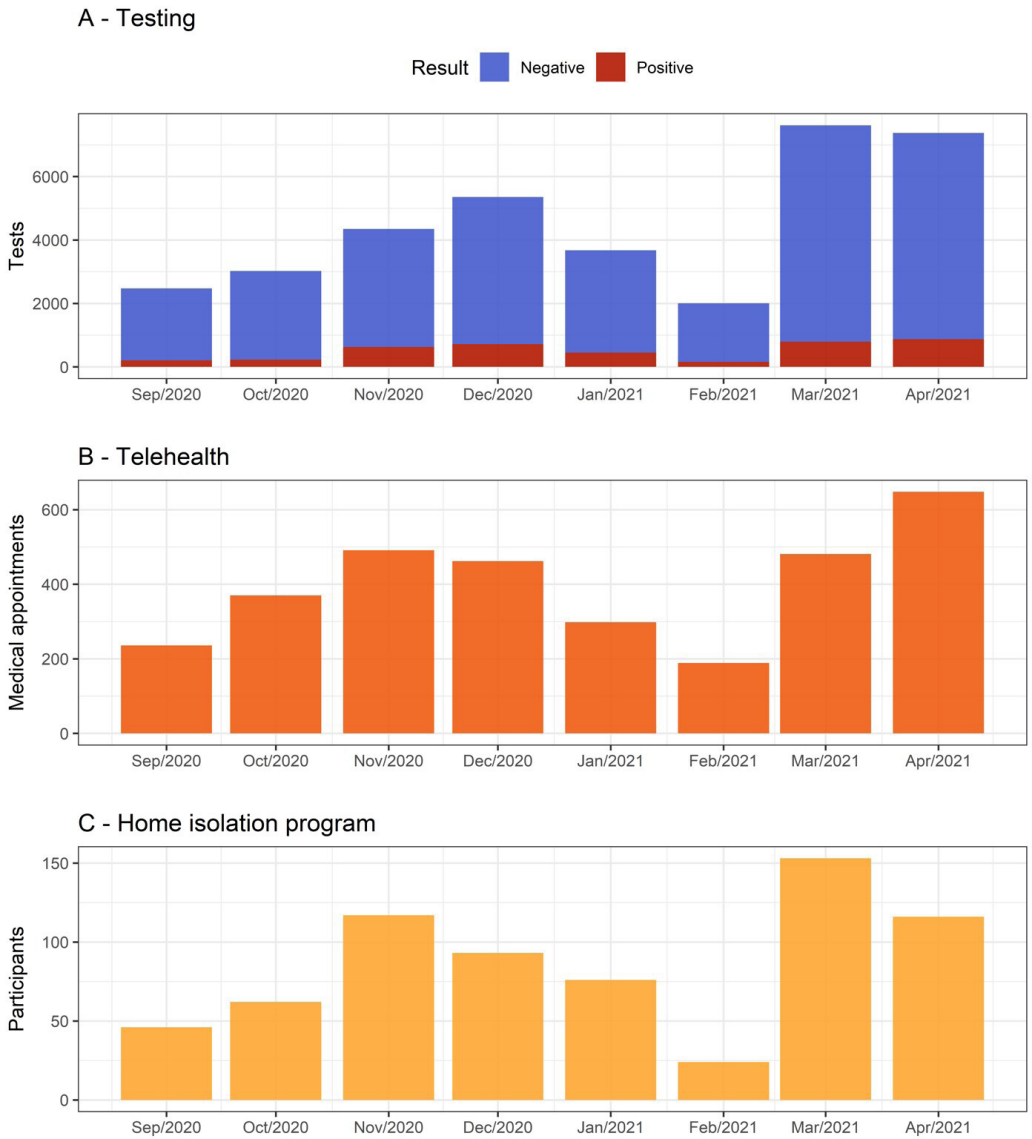
Project outputs from September 2020 to April 2021. The panel shows the total of Maré residents who attended each strategy of the initiative: Total of (A) RT-PCR tests performed, (B) telemedicine appointments and (C) isolated participants stratified by month.

We evaluated the performance of each component’s strategy at the end of the intervention period. During the intervention, 29 592 Reverse transcription PCR (RT-PCR) tests (online supplemental table E) were performed on *Maré* residents (213 tests per 1000 population), and 3478 out of these (11.7%) were positive at the end of the period (figure 4A). The number of positive tests originating from the testing strategy of our multifaceted intervention corresponded to 97.5% (3478/3569) of the total reported cases by the municipality’s monitoring system during the period for this region. Most of the positive cases were women (61%), aged between 30 years and 49 years (47%), and black and brown self-reported race (64%). In addition, no differences were identified between the demographic characteristics of the positive and negative groups (online supplemental table E).

Overall, 5577 telehealth consultations were performed, mainly focusing on psychology, nursing and medical visits. The intervention conducted 3175 online clinical appointments from September 2020 to April 2021 (figure 4B). There was also significant demand for psychological appointments (1478 patients).

The isolation programme provided reception, care and follow-up to 747 residents with COVID-19 (figure 4C). Before they received a positive result from the RT-PCR test, a social worker interviewed them to identify the need of each individual and their family. Thereby, 598 cleaning and protection kits were delivered to families during the period analysed. In addition, food insecurity was one of the main demands reported by the participants. Besides, the initiative distributed food kits to 211 families, who received three meals daily (lunch, snacks and dinner). At the end of the quarantine, 733 (98%) of the residents assisted by the programme considered that the supplies offered were essential to guarantee isolation, and 613 (87%) maintained isolation for more than 14 days.

## Discussion

Here we demonstrated the impact of a multicomponent intervention on reducing COVID-19 deaths in a socially vulnerable urban community from a tropical metropolis in a middle-income country highly affected by the burden of COVID-19. The intervention significantly increased the detection of new cases through the availability of free testing inside the community and the communication strategies inviting people to test. Additionally, there was a significant reduction in case fatality rates after the intervention started compared with *favela*s with equivalent sociodemographic profiles in the same metropolitan area. This study indicates that a community-based multicomponent intervention, using digital technologies such as mobile contact tracing and testing, and telehealth, combined with multiple community engagement strategies, may reduce the impact of COVID-19 on vulnerable communities in a middle-income country.

Socioeconomic vulnerabilities affect the number of COVID-19 cases, mortality, and access to healthcare services or testing^32^ in distinct places such as Geneva,^33^ San Francisco^34^ or Mexico City,^35^ among others. In Brazil, socioeconomic inequalities disproportionately affected the evolution of the pandemic and the outcome for more vulnerable populations.^36^ Our findings confirmed that the people living in *favelas* were more severely affected at the beginning of the pandemic than the city’s average.^17 36^ Before the intervention, Maré had worse indicators than the control group or the municipality, reporting more COVID-19 deaths per 100 000 population and a higher case fatality rate. Although the impact of social determinants was evident in case progression and outcome, health authorities or governments gave little attention or even no decisive actions were taken to minimise the effects of these inequities since the beginning of the pandemic. These facts are evident in rich countries like the USA^37^ or Switzerland,^33^ where poorer neighbourhoods had less access to testing.

An integrated intervention combining different actors (the academy, non-governmental organisations and the government) to expand information, surveillance, access to free tests and better case management proved effective, increasing case detection and reducing mortality. However, the epidemiological scenario evolved during the intervention period, with better detection of cases, and a reduction in the case fatality rate for the city of Rio de Janeiro, due to improved surveillance, including more accessible testing per inhabitant.^38^

Using DID, a causal modelling approach to estimate the effects before and after an intervention, we demonstrated that the multicomponent intervention reduced by 44% the number of reported death rates per week in *Maré*. In addition, 36% more cases per inhabitant were detected after the intervention. These improvements may be explained by the increased information and demand for testing, with more accessible tests, promoting community engagement and, therefore, expanding the notification of cases. These findings support the effectiveness of a multicomponent intervention in developing pandemic surveillance and improving access to care and outcomes in a vulnerable community.

The strategies of community engagement and communication were fundamental for the results obtained by the intervention. The involvement of *Redes da Maré*, a local organisation with more than 20 years of working with the community, was essential to the project. Their knowledge about the territory and the use of appropriate language might have contributed to the population’s acceptance of the actions proposed. In addition, the use of a data-based approach to provide information about the progression of the pandemic, clarify doubts, and identify myths and fake news, may have facilitated resident trust and adhesion. It is important to note that communication strategies were developed considering physical and digital products.

The pandemic accelerated the incorporation of digital technologies in healthcare by adapting or developing new solutions to support people during the pandemic.^39^ The use of digital solutions and innovative approaches, from machine learning applications to community networks, contributed to health surveillance in several countries.^40 41^ Digital solutions to support health surveillance have been sparse in middle-income countries, mainly in communities exposed to social vulnerability. Vulnerable populations have already faced several barriers to accessing digital technologies and have quickly been left behind.^42^ Apparently, there was no overall resistance to the community’s adherence to the intervention. However, we observed a higher proportion of working-age women seeking COVID-19 tests and difficulties reaching specific populations (eg, the elderly, children, men, etc). The mass testing strategy associated with the active participation of residents might be one of the reasons for the success of the intervention since, during several periods of the pandemic, access free of charge to RT-PCR COVID-19 testing was limited in Brazil.^43^

The combined use of telehealth and remote monitoring could have improved case management, especially in a community with limited access to healthcare. Additionally, many telehealth consultations were performed during the intervention with high acceptance by the users.^26^ Previous studies suggested remote monitoring and continuous pulse oximetry were associated with reduced mortality in COVID-19.^44 45^ The voluntary social isolation of people living in vulnerable conditions significantly impacts the families’ income.^36^ We observed that most patients with COVID-19 who agreed and complied with self-isolation were due to the food support and orientation guaranteed by the intervention.

We demonstrated that a complex intervention in a poor urban community could increase case detection and reduce mortality. The intervention’s active community engagement and communication component, combined with mobile technologies, improve access to testing and case management. We believe these results might support future actions to establish a more equitable pandemic response, especially regarding access to the health system and care for socially vulnerable populations.

The findings of this study have to be seen in light of some limitations. First, we could not distinguish the individual effects of each strategy implemented. Complex interventions require specific implementation evaluation, much limited by pandemic periods, such as the adherence to particular components and outcomes at the individual level, including a mixed-methods study.^46^ Second, it might have some concurring interventions occurring in the three control favelas in this ecological design. However, Brazil did not implement a coordinated national policy for contact tracing or community case management during the study period,^47^ but other social actions and support might have occurred.^48^ Hence, the results found in this design may be underestimated if the concurrent interventions did not have unintended consequences. Third, there might be underdiagnosis and under-reporting of COVID-19 cases due to the limited availability of tests and the overload in the city’s health system. However, we do not expect to affect the notification of COVID-19 deaths. All data considered for comparison came from the same official sources. Fourth, there may be a spatial heterogeneity related to the participation by residents since those residents located near the testing centre have easier access and more information about the intervention than the more distant ones, similar to previous studies about participatory interventions in other areas of public policy in Brazil.^49^ Finally, the social organisation (*Redes da Maré*) involved in the local actions has been working in *Maré* for several years. Thus, it is unclear whether it would be possible to replicate this finding in other areas without a solid local organisation.

## Conclusion

In conclusion, the integrated health surveillance and care model to support Rio de Janeiro’s *favelas* during the pandemic was capable of preventing deaths and improving case detection and management. This initiative guaranteed protection to a socially vulnerable population by reducing the impact of inequities in access to healthcare through the promotion of effective local actions.

## Data Availability

The datasets generated and analysed during the study are not publicly available but may be available from the corresponding author at a reasonable request. Data aggregated from third-party sources must be requested directly from the source institution.
