# Supplementary material for: Effectiveness of a multicomponent intervention to face the COVID-19 pandemic in Rio de Janeiro’s favelas: difference-in-differences analysis
Source: BMJ Glob Health. 2023 May 30;8(5):e009997. doi: 10.1136/bmjgh-2022-009997 (PMC10230340; doi:10.1136/bmjgh-2022-009997)
Supplement: Supplementary data [file bmjgh-2022-009997supp001.pdf]

Effectiveness of a multi-component intervention to face the COVID-19 pandemic in Rio de Janeiro's Favelas: Difference-in-Differences analysis

Supplementary data

Contents

Text. Institutions responsible for the multi-component intervention 2

Supplementary Method (sMethod). Difference-in-differences analysis 3

Table A. Socioeconomic indicators 4

Table B. Relative change of pandemic indicators comparing before and after the intervention for each favela in the control group (Rocinha, Cidade de Deus, Mangueira) 5

Table C. Comparison of Age group and Sex distribution between intervention group (Maré) and the control group 6

Table D. Demographics of COVID-19 deaths reported in the intervention (Maré) and the control groups (Rocinha, Cidade de Deus, Mangueira) comparing before and after the intervention periods 7

Table E. Demographics of Maré residents tested in the community from September 2020 to April 2021 8

Text. Institutions responsible for the multi-component intervention

Fundação Oswaldo Cruz (Fiocruz): Fiocruz is the most traditional research institution on infectious diseases in Latin America, with more than 120 years of activity. Fiocruz works in research, production of inputs (vaccines, diagnostic kits, and medicines), human resources training, and innovation. Additionally, Fiocruz has several reference laboratories for infectious diseases and technological platforms that support research and innovation. More than 100 million vaccine doses were produced in 2019, and over 300,000 tests performed in reference laboratories. Fiocruz also has 1,700 doctors on staff and 323 research lines registered. Fiocruz has historically supported projects in favelas and vulnerable communities, which was intensified during the COVID-19 pandemic.

Redes da Maré: Redes da Maré is a civil society institution that produces knowledge, projects, and actions to ensure adequate public policies to improve the lives of 140,000 residents of Maré's 16 favelas. Redes da Maré works to increase the quality of life, in an attempt to guarantee the fundamental rights of Maré population.

Saúde, Alegria e Sustentabilidade Brasil (SAS Brasil): SAS Brasil is a nonprofit health institution created in 2013 that, in response to the COVID-19 pandemic, was offering clinical and psychological care through teleconsultations, targeting low-income populations. The SAS telemedicine project began in March 2020, involving more than 430 healthcare volunteers, distributed among 22 different medical and seven nonmedical specialties, and also performed remote consultations through their own system.

Dados do Bem (DdB): DdB is an epidemiological monitoring project that brings together geolocation technology and methodology for real-time follow-up coronavirus evolution in urban centers. The tool generates a virus distribution map and strategic data about Covid-19 for decision-making by the authorities. Initially developed as part of a research, DdB was created by infectious disease specialists and an intelligence team, provided free of charge to the government.

Conselho Comunitário de Manguinhos: Conselho Comunitário de Manguinhos is a neighbourhood council that aims to contribute to the sustainable development of the communities around Manguinhos. It is an autonomous body that promotes actions and debates between residents, private, governmental, and socio-community institutions.

União Rio: União Rio is a voluntary movement of civil society in Rio de Janeiro that brings together people, companies, and non-governmental organizations to preserve lives. They raise the main demands in the health area and other issues concerning vulnerable communities in order to reduce the impacts of the Covid-19 pandemic.

Supplementary Method (sMethod). Difference-in-differences analysis

Missing values: To describe patients' clinical characteristics, outcomes, and organ support, we provided their corresponding number of complete cases for incomplete variables. No imputation method was performed.

Difference-in-differences (DID) modeling: Our data comprised weekly rates of reported cases and deaths in the intervention group (Maré) and the control group (a combination of Rocinha, Cidade de Deus, and Mangueira).

To estimate the effect of the multi-component intervention, we obtained the classic difference-in-difference estimator using a Negative Binomial regression model.

Outcome: Reported number of cases and deaths per 100,000 population per age and sex

Multivariable Negative binomial regression model: As our outcomes are rates, defined as the ratio between a count variable (number of events) and a denominator (population), we modeled them using a Negative binomial distribution assumption in the regression. The multivariable model syntax is defined as:

$Outcome_{age\_strata}$ *(count) ~ Intervention/Control indicator + Period indicator + Intervention/Control indicator * Period indicator + Age group + Sex + offset(log(*$population_{age\_strata}$*))*

This model syntax means that the outcome (number of events) was explained by the indicator of the intervention (Maré) or control group, the period of intervention onset (before or after), an interaction term between the groups and the period, the age, and the sex groups. We included the log(population) as an offset variable (slope = 1) to model the outcome's denominator for the rate.

The DID estimator corresponds to the coefficient of the interaction term (intervention group * period). However, in the Negative Binomial regression, this estimate is in log scale. Hence, we obtained the DID estimator as the Rate of Rate Ratios (RRR), defined as the exp(estimate).

Table A. Socioeconomic indicators

	Rio de Janeiro Capital	Intervention group (Maré)	Control group (Rocinha, Cidade de Deus, Mangueira)	Rocinha	Cidade de Deus	Mangueira
**Population**	6,320,446	129,770	123,706	69,356	36,515	17,835
**Population density (person / km²)**	15,816	73,090	68,073	79,031	35,283	25,094
**Age group, years**						
0 – 9	759,791	21,260	20,264	11,349	5,743	3,172
10 – 19	930,717	23,473	21,980	11,876	6,747	3,357
20 – 29	1,059,810	25,746	25,422	15,852	6,222	3,348
30 – 39	992,986	22,951	20,629	12,659	5,456	2,514
40 – 49	876,487	16,514	15,602	8,315	5,134	2,153
50 – 59	759,804	10,800	10,531	5,404	3,413	1,714
60+	940,851	9,026	9,278	3,901	3,800	1,577
**Sex**						
Female	3,360,629	66,027	63,943	35,144	19,326	9,473
Male	2,959,817	63,743	59,763	34,212	17,189	8,362
**Income per person***	$ 165.33	$79.16	$84.19	$81.76	$89.78	$82.16
**SPI**	0.609	0.547	0.541	0.533	0.559	0.537
**HDI index**	0.771	0.686	0.660	0.663	0.67	0.628
**HDI education**	0.673	0.510	0.481	0.461	0.543	0.429

*$1.00 = R$ 4.99

Table B. Relative change of pandemic indicators comparing before and after the intervention for each favela in the control group (Rocinha, Cidade de Deus, Mangueira)

Local	Before intervention (Mar/2020 – Sep/2020)	After intervention (Sep/2020 – Apr/2021)	Relative Change
**Rocinha**			
Cases	952	1872	97%
Standardised cases per 100,000	84	99	18%
Average Standardised cases/100,000 per week*	104 (27.0)	109 (23.0)	5%
Deaths	80	45	-44%
Standardised Deaths per 100,000	7	2	-71%
Average Standardised deaths/100,000 per week*	15 (8.1)	5 (2.3)	-67%
Standardised Case-fatality ratio	8.9%	3.3%	-62%
**Cidade de Deus**			
Cases	375	704	88%
Standardised cases per 100,000	96	100	4%
Average Standardised cases/100,000 per week*	99 (15.7)	103 (13.2)	4%
Deaths	79	75	-5%
Standardised Deaths per 100,000	20	11	-45%
Average Standardised deaths/100,000 per week*	23 (7.6)	15 (3.9)	-35%
Standardised Case-fatality ratio	18.1%	9.3%	-49%
**Mangueira**			
Cases	201	362	80%
Standardised cases per 100,000	151	152	1%
Average Standardised cases/100,000 per week*	154 (27.0)	156 (21.0)	1%
Deaths	29	16	-45%
Standardised Deaths per 100,000	22	7	-68%
Average Standardised deaths/100,000 per week*	24 (12.2)	9 (5.4)	-63%
Standardised Case-fatality ratio	15.6%	4.2%	-73%

*Mean (SD)

Table C. Comparison of Age group and Sex distribution between intervention group (Maré) and the control group

	Intervention group (Maré)	Control group (Rocinha, Cidade de Deus, Mangueira)	SMD
**Age group, years**			0.037
0 – 9	21,260 (16.4%)	20,264 (16.4%)	
10 – 19	23,473 (18.1%)	21,980 (17.8%)	
20 – 29	25,746 (19.8%)	25,422 (20.6%)	
30 – 39	22,951 (17.7%)	20,629 (16.7%)	
40 – 49	16,514 (12.7%)	15,602 (12.6%)	
50 – 59	10,800 (8.3%)	10,531 (8.5%)	
60+	9,026 (7.0%)	9,278 (7.5%)	
**Sex**			0.016
Female	66,027 (50.9%)	63,943 (51.7%)	
Male	63,743 (49.1%)	59,763 (48.3%)	

SMD: Standardised Mean Differences

Table D. Demographics of COVID-19 deaths reported in the intervention (Maré) and the control groups (Rocinha, Cidade de Deus, Mangueira) comparing before and after the intervention periods

	**Intervention group (Maré)**				**Control group (Rocinha, Cidade de Deus, Mangueira)**			
Characteristic	Overall, N = 279[1]	Before intervention, N = 199[1]	After intervention, N = 80[1]	Relative change	Overall, N = 324[1]	Before intervention, N = 188[1]	After intervention, N = 136[1]	Relative change
**Age group, years**								
0-39	28 (10.0%)	20 (10.1%)	8 (10.0%)	-60%	24 (7.4%)	11 (5.9%)	13 (9.6%)	18%
40-59	76 (27.2%)	52 (26.1%)	24 (30.0%)	-54%	84 (25.9%)	52 (27.7%)	32 (23.5%)	-38%
60+	175 (62.7%)	127 (63.8%)	48 (60.0%)	-62%	216 (66.7%)	125 (66.5%)	91 (66.9%)	-27%
**Sex**								
Female	130 (46.6%)	83 (41.7%)	47 (58.8%)	-43%	174 (53.7%)	95 (50.5%)	79 (58.1%)	-17%
Male	149 (53.4%)	116 (58.3%)	33 (41.2%)	-72%	150 (46.3%)	93 (49.5%)	57 (41.9%)	-39%

[1]n (%)

Table E. Demographics of Maré residents tested in the community from September 2020 to April 2021

Characteristic	Overall, N = 29,592[1]	Positive, N = 3,478[1]	Negative, N = 26,114[1]
Sex			
Female	18,612 (63%)	2,124 (61%)	16,488 (63%)
Male	10,980 (37%)	1,354 (39%)	9,626 (37%)
Age group, years			
<20	2,214 (7.5%)	280 (8.1%)	1,934 (7.4%)
20-29	5,766 (19%)	648 (19%)	5,118 (20%)
30-39	6,802 (23%)	888 (26%)	5,914 (23%)
40-49	6,194 (21%)	744 (21%)	5,450 (21%)
50-59	4,888 (17%)	552 (16%)	4,336 (17%)
60+	3,728 (13%)	366 (11%)	3,362 (13%)
Self-reported race			
Black or Brown	16,414 (65%)	1,950 (64%)	14,464 (65%)
White	8,200 (32%)	1,016 (33%)	7,184 (32%)
Asian	588 (2.3%)	58 (1.9%)	530 (2.4%)
Indigenous	134 (0.5%)	10 (0.3%)	124 (0.6%)
NA	4,256	444	3,812
Healthcare workers	1,978 (6.7%)	200 (5.8%)	1,778 (6.8%)
Previous positive test result	2,792 (9.4%)	158 (4.5%)	2,634 (10%)
NA	2	0	2

[1]n (%)
